# Predictors of weight loss in young adults who are over-weight or obese and have psychosocial problems: a post hoc analysis

**DOI:** 10.1186/s12875-016-0437-8

**Published:** 2016-04-11

**Authors:** Jørgen Lous, Kirsten S. Freund

**Affiliations:** Research Unit of General Practice, Department of Public Health, University of Southern Denmark, J.B. Winsløwvej 9A, DK-5000 Odense, Denmark; General Practice, DK-9362 Gandrup, Denmark

**Keywords:** Weight loss, Life coaching, General practitioners, Over-weight, Psychosocial problems, Specific self-efficacy, Patient-centred, Motivation

## Abstract

**Background:**

The aim of this study is in a general practice trial setting to identify predictive factors for weight loss after 1 year among young adults who are over-weight or obese and who have several psychosocial problems.

**Methods:**

Twenty-eight general practitioners recruited 495 patients aged 20–45 years with psychosocial problems for a randomized general preventive study to increase self-efficacy to achieve a self-prioritised goal for a better life by discussions of resources and barriers for reaching the goal. The present study is a post hoc analysis of possible predictors of weight loss among all 218 patients who have over-weight or obesity. A 23-pages questionnaire was completed before and 1 year after randomization. 111 patients had a one-hour preventive health consultation with their general practitioners focused on life coaching and a follow-up consultation within 3 months, and 107 patients had no preventive consultation.

**Results:**

Twenty-two patients stated during the preventive consultation that weight loss was a prioritised goal. They had a mean weight loss of 4.7 kgs compared with 1.6 kgs in the group without this goal and 1.6 kgs in the group without preventive consultation. In a logistic regression model, predictors of weight loss or no weight loss were a) pre-interventional consideration of weight loss within 30 days, b) having weight loss as a prioritised goal for improved quality of life, c) being female, d) being in the oldest half of participants, and e) having many psychosocial problems. In a linear regression model, the predictors together explained about 11 % of the weight loss. Important predictors were: obesity (explained 4 %), pre-interventional consideration of weight loss within 30 days (3 %), and having a preventive health consultation with weight loss as a prioritised goal (2 %).

**Conclusions:**

Pre-interventional consideration of weight loss within 30 days and having weight loss as a prioritised goal during the health consultation were two important predictors for weight loss. By structured interventions focussing on the patients’ priorities, self-chosen goals, their resources and barriers for reaching the goals, changes may be obtained; especially in participants with many problems who often do not accept participation in procedures on risks.

**ClinicalTrials gov Registration:**

NCT 01231256, Aug. 22. 2010.

## Background

Excess bodyweight is an important risk factor for mortality and morbidity, causing nearly 3 million deaths every year worldwide [1]. Globally, body mass index (BMI) has increased since 1980 with large differences between nations and regions [1, 2]. A recent meta-analysis found that obesity, especially with BMI ≥35 kgs/m^2^, was associated with significantly higher all-cause mortality, and slight overweight was associated with significantly lower all-cause mortality [3].

The importance of overweight (BMI 25–30 kgs/m^2^) and obesity (BMI >30 kgs/m^2^) as risk factors for myocardial infarction and ischemic heart disease is controversial. A register-based cohort study including all patients from The Western Denmark Heart Registry with coronary atherosclerosis confirmed via coronary angiography (*n* = 37 573) found that the death rate was lowest among pre-obese patients (BMI 25–30 kgs/m^2^) during 11 years of follow-up [4]. A Danish cohort study of 71 527 individuals from the Copenhagen General Population Study with 3.6 years of follow-up reported increased risk of myocardial infarction in overweight (hazard ratio 1.26 (95 % confidence interval (CI) 1.0 to 1.6)) and obese (hazard ratio 1.88 (1.3 to 2.6)) subjects [5].

Many short-term studies have evaluated the effects of weight loss in patients with chronic diseases and often demonstrated a beneficial effect of even modest weight loss on their disease [3, 6]. Fewer long-term studies have evaluated the benefits of weight loss, but their findings tend to confirm the findings of short-term studies [7, 8]. In Denmark, overweight and obesity are significant problems increasing with age [9, 10].

While some consequences of obesity are well documented, the reasons for over-weight and obesity are very complex and difficult to address. The importance of human gut microbes is under intense investigation [11]. Imbalance between energy input and energy use is, however, part of the problem and is the focus of many interventions programmes. Intervention with dietary advice, more exercise, and improved health professional management has resulted in effects that are often limited and brief [12–14]. In contrast, focusing on self-efficacy, which is a person’s judgement of his or her ability to cope effectively in a specific difficult situation, seems to result in a better change of life style over 6–12 months [15, 16].

People with mental health problems often have over-weight problems as well. Several studies have shown positive effects of health promotion coaching resulting in clinical significant weight loss and an increase in the sense of coherence [17, 18].

Approximately 90 % of the Danish population, visit their general practitioners (GPs) at least once annually [19]. Thus it is evident that Danish GPs may play a central role in treating over-weight and obesity in their surgeries.

In 1998–9 we did a randomized study on the effect of patient-centred consultations in general practice for 20–44 year old patients with multiple psycho-social and lifestyle problems [20]. The focus was on resources and barriers for obtaining self-chosen goals within life circumstances and lifestyle. One-year follow-up questionnaire showed significant improvement in Short Form Health-related Quality of Life Mental (MCS-SF12) in the intervention group (7.3 point) compared with the control group (3.0 point). The difference was 4.3 point (ci 1.6 to 7.0, *P* = 0.002). The number of problems were reduced significantly more in the intervention than in the control group (1.8 versus 0.8, *p* = 0.03) [20].

This paper is a post hoc analysis of all 218 patients who are over-weight or obese, irrespective of their randomization group. In the previous paper, we found that the intervention group (independent of their weight) had a mean weight loss of 2.9 kgs compared with the control group weight loss of 1.5 kgs, (difference 1.4 kgs (ci: −0.8 to 3.7 kgs, *p* = 0.21) [20].

The primary aim of the present paper is to describe predictive factor for weight loss in participants with a BMI at 25 or higher. Our secondary aim is to describe the participants who are over-weight or obese compared with the participants in the normal weight group.

Weight loss was the most common chosen goal for a better life next year independent of weight. Other chosen goals were better psychological health, a better relationship to the partner, a change of job situation, smoking cessation, better health, and a better social network [20].

## Methods

### GPs

All 325 GPs in North Jutland County, Denmark, were invited to participate. Fifty (15 %) attended the study introduction weekend session, and 28 GPs included patients in the study. They had a total of 40 h of preventive health education, partly weekend, partly one-day or evening sessions concentrating on psycho-social factors, lifestyle problems related to cardiovascular disease, alcohol, smoking, and drug addiction, the “stages of change” model, patient-centred consultation with important elements from motivational interviewing respecting patients’ goals and ambivalence, discussing and supporting patients’ own resources and barriers to achieve the patients’ goals [20].

### Material

Participants were recruited by the secretary when attending the GPs’ surgery for other reasons. They were required to be 20–45 years old, able to read and understand Danish, and without any severe acute illness or severe psychiatric problems. A total of 2 056 people (98 %) accepted the invitation to participate in the preventive study and provided written informed consent about the purpose:”… To support your resources in order to prevent larger problems or illness…”. They were screened by completing a “problem questionnaire” with 33 items about self-rated health, personal network and resources, lifestyle, and social situation [18]. A cut-off of ≥7 of 33 problems was chosen in order to include the quarter of participants with the largest number of problems, actually 625 (30 %) had ≥7 problems [21]. These 625 individuals were invited to participate in the randomised trial.

All participants had to complete a baseline questionnaire at home consisting of 80 questions (23 pages) dealing with family situation, resources, work, education, self-rated health, use of medicine, dietary and smoking habits, substance abuse, height and weight, health-related quality of life Short Form (SF12), health and illness behaviour and considerations of changes for improved quality of life the following year. A total of 495 participants returned the baseline questionnaire and were randomised to intervention or control groups regardless of their weight. All 495 were sent a postal 1-year follow-up questionnaire (23 pages) similar to the baseline questionnaire.

### Intervention

Participants in the intervention group were invited to attend a one-hour preventive health consultation as well as a 20-min follow-up consultation with their GPs within 3 months. Completing the baseline questionnaire was intended to facilitate insight into circumstances regarding psychosocial life, health, lifestyle, the participant’s reaction to stressors, and heath and illness behaviour. This insight made it easier for the GPs to offer patient-centred counselling [20]. The GPs were recommended to use open questions and to respect the patients’ agendas by asking the following questions: “What was it like to complete the questionnaire?” and “What do you prefer to discuss?” During the health consultation, the patients were asked to choose 1 or 2 goals to improve their quality of life the following year. At the end of the health consultation, the patients’ resources and barriers for achieving their goal were discussed and described, and their time schedules were written down. This style of intervention is now called life coaching [22].

### Possible predictors of weight loss

The study is based on the following information: 1) the screening questionnaire and baseline questionnaire completed by all 495 participants before randomisation, 2) the goal chosen during the health consultation, and 3) 1-year follow-up questionnaires including their actual height and weight, returned by post.

Demographic variables such as age, gender, education, cohabitation status, self-rated economy (bad to fair/good), and social group were included in the baseline questionnaire as were self-rated health, Short Form Health-related Quality-of-Life Mental (MCS-SF12) and Physical (PSC-SF12), and the number of problems on the 33-item screening questionnaire [21].

### Statistics

Data were collected from the questionnaires using the TeleForm^R^ reading system (info@cardiff-teleform.com) and analysed in IBM SPSS (Statistical Package for the Social Sciences, ver. 16 and 22). Scaled variables were analysed both as scaled and as far as possible dichotomised into two equal-sized parts. The dichotomizing was done with biological meaningful cut points. Dichotomizing means fewer cells in the analysis and a more stable model with our number of cases. Achieved 1) weight loss (yes or no) and 2) extent of weight loss after 1 year were the two dependent variables. Unadjusted analyses were performed using simple descriptive statistics such as Fisher’s exact test, the chi-squared test, odds ratios (ORs) and analysis of variance (ANOVA) with 95 % confidence intervals (ci). Adjusted analyses in logistic regression models for weight loss or no weight loss as well as linear regression of the size of weight changes after 1 year were done with age group, gender, and variables that showed significant relation to weight loss in unadjusted analyses or a *p*-value less than 0.20. All non-over-weight respondents were excluded from the predictor analysis. All *p*-values are two-sided, and *P* < 0.05 was considered to indicate statistical significance.

### Ethics

The Scientific Regional Ethics Committee in North of Jutland, now called North Denmark Region and the Danish Data Protection Agency approved this study. Patients were informed by their GPs, received written information, and provided written consent to participate in the study. The study protocol for the randomised controlled trial was published in ClinicalTrials.gov under registration number NCT 01231256.

## Results

### Participants

**Fig. 1:**
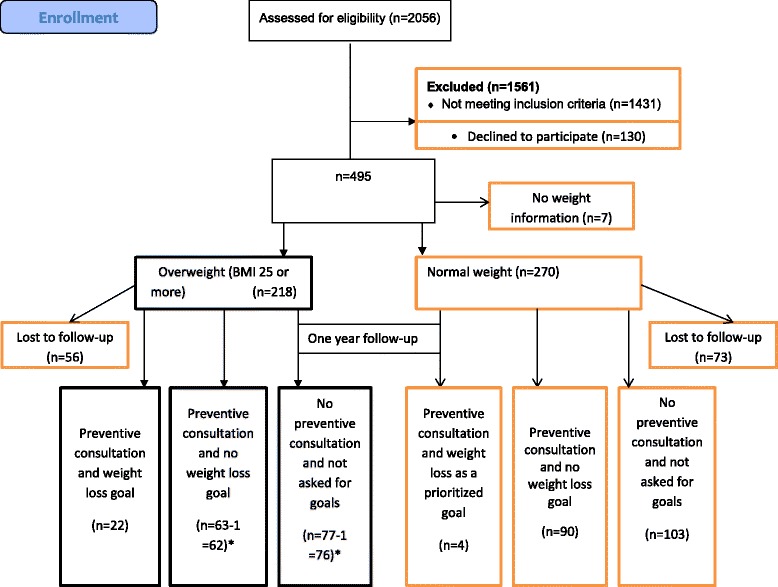
Flowchart of enrollment. Note: one participant excluded because of missing information on weight both in the group of preventive consultation and no weight loss goal and in the group of no preventive consultation and not asked for goals

A total of 218 respondents with ≥7 psychosocial and lifestyle problems and who are over-weight (BMI 25–30 kgs/m^2^) or obese (BMI 30–54 kgs/m^2^) were included (Fig. 1). Over-weight was reported by 128 and 90 reported obesity. A total of 270 (55 %) of the randomised 495 had normal weight. Seven (2 %) had not reported height or weight at baseline, which is why they were excluded. Fifty-six (26 %) of the 218 obese/over-weight participants were lost to follow-up after 1 year, and two had missing value of weight at 1-year follow-up, leaving 160 with over-weight for the predictor analysis (Fig. 1). No difference between attenders and drop-outs was found, and drop-out rate was not related to baseline weight.

### Measured and stated weight

**Fig. 2:**
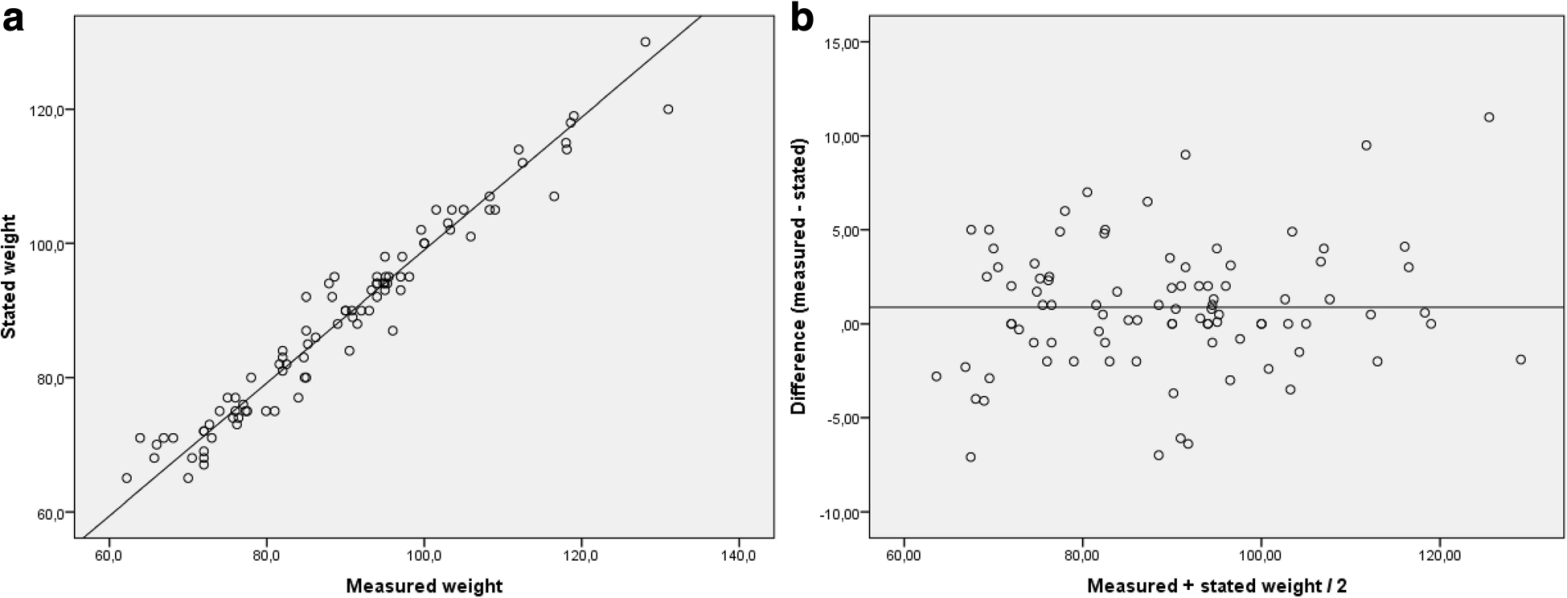
**a** and **b** Correlation plot and “Difference versus average plot” (Bland & Altman plot) of measured and stated weight at baseline (*n* = 91)

All 218 with over-weight had self-reported measurements of weight and height at baseline. In the intervention group, we could analyse the difference between measured weight during the first consultation and reported weight in the baseline questionnaire delivered to the GPs before randomisation. An agreement analysis was done with a correlation plot and a Bland-Altman plot (difference versus average plot) [23]. Spearman’s rho of 0.97 and a difference of 0.89 kg (ci 0.2 to 1.6 kgs) were found (Fig. 2). These agreements justify comparison of stated weight at baseline and after 1 year in this study.

### Characteristics of the over-weight group

The group of over-weight and obese patients (over-weight group; *n* = 218) had a mean weight of 88.6 kgs and a mean BMI of 30.2 kgs/m^2^; the baseline values for the non-over-weight group (*n* = 270) were 63.2 kgs and BMI of 21.8 kgs/m^2^, respectively (Table 1). Over-weight patients were more often male (OR = 1.40, ci 1.04 to 1.9) and were approximately 1 year older (34.7 years old) than participants with normal weight (33.5 years old). Educational background, self-rated health, and number of problems were not significantly different. The over-weight group had a lower physical health-related quality of life (PCS-SF12) (ANOVA, *P* = 0.038) and the same mental health-related quality of life as the non-over-weight group (MCS-SF12) (Table 1).

**Table 1:** Baseline characteristic of over-weight or obese compared with no over-weight participants (*n* = 488)

Baseline characteristic	Overweigth		No overweigt		Anova or Relativ Risk (95 % conf interv)
	*n* = 218		*n* = 270		
Weight, kg, mean (95 % CI) (SD)	88.6 (86.5–90.9)	(16.4)	63.2 (62.1–64.3)	(9.2)	ANOVA, *F* = 468.2 *P* = 0.000
Height, cm, mean, (95 % CI) (SD)	171.2 (170.1–172.4)	(8.9)	170.1 (169.1–171.1)	(8.5)	ANOVA, *F* = 2.0 *P* = 0.16
BMI, kg/m^2^ ^a^, mean (95 % CI) (SD)	30.2 (29.5–30.8)	(4.7)	21.8 (21.5–22.0)	(1.9)	ANOVA, *F* = 702.5 *P* = 0.000
Age, years, mean (95 % CI)(SD)	34.7 (34.4–36.2)	(6.7)	33.5 (33.3–34.9)	(6.6)	ANOVA *F* = 3.9 *P* = 0.049
Sex, number male/female (%)	70/148	(32.1 %)	62/208	(23 %)	RR = 1.40 (1.04 to 1.9) *P* = 0.025
Not single/single (%)	160/58	(73.4 %)	186/82	(w69.4 %)	RR = 1.22 (0.82 to 1.8) *P* = 0.36
11 years or more school education, number (%)	70/148	(32.1 %)	100/170	(37.0 %)	RR = 0.87 (0.68 to 1.11) *P* = 0.44
Self-rated health	133/85	(61.0 %)	154/115	(57.2 %)	RR = 1.17 (0.81 to 1.68) *P* = 0.41
Bad to fair/good to very good, number (%)					
Consider a short term weight loss, number (%)	46/169	(21.4 %)	15/254	(5.6 %)	RR = 3.84 (2.2 to 6.7) *P* = 0.000
Number of problems, mean (95 % CI) (SD) (min 7 and max 33)	9.9 (9.6–10.3)	(2.9)	10.3 (10.0–10.7)	(3.1)	ANOVA, *F* = 2.1 *P* = 0.15
Mental score (mcs-SF12), mean (SD) (*n* = 216 and 262)	40.4 (38.8–42.0)	(11.9)	39.8 (38.4–41.1)	(10.7)	ANOVA, *F* = 0.40 *P* = 0.53
Physical score (pcs-SF12), mean (SD) (*n* = 216 and 262)	46.8 (45.3–48.2)	(11.0)	48.8 (47.5–50.1)	(10.5)	ANOVA, *F* = 4.31 *P* = 0.038
Intervention/control group, number (%)	111/107	(50.9 %)	128/142	(47.4 %)	RR = 1.07 (0.90 to 1.29) *P* = 0.25
Weight loss as a prioritized goal for the next year^b^ number (%)	27/191	(12.4 %)	5/265	(1.9 %)	RR = 7.49 (2.8 to 19.8) *P* = 0.000

^a^Seven had missing information of BMI

^b^Control group not asked about goals

At baseline the over-weight group more often (21 %) considered weight loss within 30 days compared to 5 % in the non-over-weight group ( RR = 3.8; Table 1). A total of 32 participants had weight loss as their top prioritised goal, and 27 of them were over-weight (relative risk = 7.49; Table 1).

### Possible predictors of weight loss in the over-weight group

In Table 2, the possible predictors for weight loss are listed with their mean weight changes (ANOVA), and their relation to weight loss or no weight loss (OR). Information is from the 160 over-weight patients who returned the 1-year follow-up questionnaire with weight information.

**Table 2:** Possible baseline predictors for weight loss after 1 year (*n* = 160)

Variable	Category	Number (160)	Mean weight change (kg)	95 % ci of the change in mean	ANOVA		OR for weight loss or not and 95 % ci
					F-value	*P*-value	
Gender	Male	48	−1.50	−3.2 to 0.2			
	Female	112	−2.21	−3.5 to −1.0	0.41	0.52	2.01
							1.01 to 4.0
Age	21 to 34 years old	64	1.72	3.4 to −0.03			
	35 to 45 years old	96	−2.19	−3.5 to −0.9	0.20	0.65	1.43
							0.8 to 2.7
Education	School 7 to 11 years	104	−2.51	−3.8 to −1.2			
	School 12 years or more	56	−1.05	−2.6 to 0.5	1.87	0.17	0.57
							0.3 to 1.1
Social group	High (1 to 4)	97	−1.75	−2.8 to −0.7			
	Low (5)	63	−2.38	−4.4 to −0.4	0.36	0.55	0.82
							0.4 to 1.6
Occupation	No occupation	38	−1.97	−4.1 to 0.1			
	Have occupation	122	−2.01	−3.2 to −0.8	0.01	0.98	0.92
							0.4 to 1.9
Single/not single	Single	39	−0.54	−2.6 to 1.6			
	Not single	121	−2.47	−3.6 to −1.3	2.68	0.10	1.50
							0.7 to 3.1
Self-rated Economy	Good, very good	48	−2.81	−5.0 to −0.6			
	Fair, bad, very bad	111	−1.45	−2.5 to −0.4	1.62	0.21	1.22
							0.6 to 2.4
Self-rated health	Good, very good	62	−1.26	−2.6 to 0.1			
	Fair, bad, very bad	98	−2.47	−3.9 to −1.1	1.35	0.25	1.96
							1.03 to 3.7
Problem group	7-9 problems (medium)	85	−1.45	−2.7 to −0.2			
	10 or more problem	75	−2.63	−4.2 to −1.0	1.34	0.25	1.98
							1.05 to 3.7
Mental QoL (SF12)	Lower half	74	−2.30	−3.8 to −0.8			
	Upper half	86	−1.74	−3.1 to −0.4	0.29	0.59	0.65
							0.3 to 1.2
Physical QoL (SF12)	Lower half	82	−2.26	−3.8 to −0.7			
	Upper half	78	−1.73	−3.1 to −0.4	0.27	0.61	0.82
							0.4 to 1.5
Overweight group	BMI 25 to 27.49	60	−0.47	−1.9 to 1.0			
	BMI 27.5 to 29.99	31	−2.03	−3.8 to −0.3			
	BMI 30+	69	−3.32	−5.1 to −1.5	3.24	0.042	1.85^a^
							0.98 to 3.5
Considered weight loss at baseline (*n* = 159)	No	20	−1.75	−3.9 to 0.4			
	Yes, within 1 year	61	−2.05	−3.6 to −0.5			
	Yes, within 6 months	41	−0.12	−2.4 to 2.1			
	Yes, within 30 days	37	−4.27	−6.5 to −2.1	2.79	0.042	3.43^b^
							1.5 to 7.9
Randomization group and weight loss goal	Control, no goal setting	76	−1.57	−3.0 to −0.12			
	Preventive consultation without weight loss as a goal	62	−1.56	−3.2 to 0.05			
	Preventive consultation with weight loss as prioritized goal for the next year	22	−4.73	−7.7 to −1.8	2.3	0.10	4.63^c^
							1.5 to 14.4

Mean size of weight loss and OR for weight loss or no weight loss in participant with BMI over 25

*ANOVA* analysis of variance; *OR* Odds Ratio; *Kg* Kilogram; *CI* Confidence interval

^a^The two weight groups below BMI 30 were collapsed to one group to calculate OR

^b^The three groups with no or longer time horizon for weight loss than 30 days collapsed to one group

^c^A new variable combining randomization and goal setting

“Having a preventive consultation and obtaining a weight loss as the first priority for better quality of life in the coming year” (OR = 4.6) and pre-interventional “consideration of weight loss within 30 days” (OR = 3.4) were the most important predictors in unadjusted analysis (Table 2). Females had an OR of 2.01 compared to males for having a weight loss, and those with low self-rated health more often achieved weight loss than those with good self-rated health (OR = 1.96). Those with 10 or more problems in the screening questionnaire more often had weight loss (OR = 1.98). The 22 participants with weight loss as a prioritised goal during their health consultation had an average weight loss of 4.7 kgs (ci 1.8 to 7.7) compared to 1.6 kgs (−0.05 to 3.2) obtained by the 62 participants without a weight loss goal and those (*N* = 76) with no preventive health consultation: 1.6 kgs (0,1 to 3.0) (Table 2).

Using backward logistic regression on predictors for weight loss or no weight loss, we evaluated the eight possible predictors with *p*-values <0.2 in Table 2 and included age group in the analysis. In this analysis (Table 3), five variables were significantly predictive of weight loss after 1 year: 1) considering a short term weight loss before the intervention, 2) weight loss as top prioritised goal for improved quality of life during the preventive consultation, 3) having many psychosocial and lifestyle problems, 4) being in the 35–45 years-old group, and 5) being female. Over-weight group did not reach significance (*p* = 0.053), but stayed in the model. Lower levels of school education, living as a single person, and self-rated health were excluded from the model (Table 3). Thus, the number of psychosocial problems pushed self-rated health out of the model.

**Table 3:** Predictors for weight loss (yes or no) after 1 year in logistic regression model (*n* = 159)

Variable in the model	Exp (B)	95 % Conf. Interval	*P*-value
Consider a short term weight loss at baseline (30 day/6 months or more)	5.80	2.2 to 15.2	0.001
Preventive consultation and weight loss as goal	0.47	0.3 to 0.8	0.005
Number of problems (10 or more/7–9)	2.87	1.3 to 6.0	0.005
Age group (35–45/21–34 years)	2.87	1.3 to 6.3	0.009
Gender (female/male)	2.52	1.1 to 5.6	0.023
Weight group (obese/overweight)	1.48	1.0 to 2.2	0.053
Constant	0.012		0.003
*Excluded from the model:*			*p*-value
School education (high/low)	2.21		0.14
Single/not single	1.07		0.30
Self-rated health (bad to fair/good)	0.73		0.39

Omnibus tests of model coefficients, Chi-square = 35.3, df = 6, *P* = 0.000

Cox&Snell R Square = 0.20, Nagelkerke R Square = 0.27

Hosmer and Lermeshow test: Chi-square = 7.2, df = 8, *P* = 0.52

To further illustrate the importance of the identified predictors, we performed linear regression analysis on the size of weight changes (Table 4). The model has a problem illustrated by the fact that the constant in the model was not significant (*P* = 0.29). For that reason, the results of the linear regression shall be read with care. The linear model supported the logistic model and explained about 11 % of the changes in weight after 1 year. Over-weight group (3.8 %), consideration of short term weight loss (3.3 %), and having a preventive health consultation, and weight loss as a prioritised goal during the consultation (2.4 %) were significant predictors of the extent of weight loss. Cohabiting (2 %) stayed in the model, but did not reach significance (*P* = 0.061). The other variables (problem group, age group, school education, self-rated health, and gender) were excluded from the model (Table 4).

**Table 4:** Predictors for the size of weight loss in a linear regression model, (*n* = 159)

Variables	Unstandardized coefficient B (95 % ci)	*p*-value	R-square change
BMI group	−1.32 (−2.4 to −0.2)	0.017	3.8 %
(25–27.4/27.5-30/30+)			
Consider a short term weight loss at baseline (30 days/6 months or more)	−3.18 (−5.5 to −0.9)	0.007	3.3 %
Preventive consultation and weight loss as a goal	1.38 (0.1 to 2.7)	0.048	2.4 %
Single/not single	−2.15 (−4.4 to 0.1)	0.061	2 %
Constant	3.29 (−2.8 to 9.4)	0.29	
Total explained by the model (R Square)			11.5 %
*Excluded from the model:*		*p*-value	R Square
Problem group (10 or more/7–9)		0.11	1.5 %
Age group (35–45/21–34 years)		0.19	0.9 %
School education (high/low)		0.26	0.3 %
Self-rated health (bad-fair/good)		0.28	0.1 %
Gender (female/male)		0.76	0 %

## Discussion

### Discussion of main results

Our investigation addressed possible predictive factors for weight loss among young over-weight and obese adults who have many psychosocial problems. One of the important factors was to have weight loss as a top prioritised goal for a better health next year and to have discussed resources and barriers for reaching the goal with their GPs during preventive consultations focusing on specific self-efficacy and life coaching. Those participants experienced an average weight loss of 4.7 kgs compared with 1.6 kgs obtained by the rest of the over-weight or obese patients (Table 2). Almost the same weight loss was experienced by the 37 who had pre-interventional consideration of weight loss within 30 days (4.3 kgs). Other important predictors were having many psychosocial problems, and being 35 to 45 years old and being female. Being obese was less important in the logistic regression model (Table 3).

At baseline, over-weight/obese participants were approximately 1 year older, were more often men, and had lower physical health-related quality of life (PCS-SF12) than members of the non-overweight group.

###  Limitations

Our participants were invited to participate in the GPs’ clinics if they were between 20 and 44 years old and had ≥7 psychosocial and lifestyle problems out of 33 possible. Thus our results cannot be generalised to the average patient population, but only to the 30 % of younger patients with several psychosocial problems coming to the clinic for any reason [20, 21].

This study is a post hoc analysis of a previous study, but with another focus than the RCT, namely predictive factors for weight loss after 1 year in those who are overweight or obese, independent of their group in the RCT.

Our analysis is also limited by our reliance on self-reported height and weight data. The completed baseline questionnaire with height and weight was delivered to each participant’s GP, and therefore a certain degree of credibility could be expected. The intervention group had their height and weight measured with good agreement (mean difference of 0.9 kg) in the Bland-Altman plot (Fig. 2b). In a web-based treatment program in the United States, self-reported weight correlated significantly with measured weight, with a difference of ~1 kg [24].

The study dropout of 56 over-weight patients out of 218 (27 %) is problematic, but it was expected in this group of young adults with several psychosocial problems. This age group moves to other regions of Denmark with a high frequency. A previous systematic review reported a mean dropout rate from lifestyle intervention programs for over-weight and obese infertile women of 24 % in 10 studies [25]. That rate is very close to our loss to follow-up.

The relatively small number of over-weight with weight loss as top prioritised goal in our analysis is a problem, which means, wide confidence intervals and limited precision in the linear regression analyses. Weight loss was the most frequently chosen goal (*n* = 33, 16 %) among the 209 patients having a health consultation.

In the statistical analysis, we dichotomised some variables. We know the problems with dichotomizing variables, primary loss of information, and the problem as to where to place the cut point. We tried to cut in a biological meaningful way that means trying to divide the material in a strait forward way or using the median as cut point. The dichotomizing meant a reduction of cells in regression analyses, and thus a more stable model.

Another limitation of your design could be our definition of “many” psychosocial problems. It could be problematic just to add up different problems of different character and importance. The reason for using this pragmatic method was that we just wanted to select persons with multiple problems. That meant problems within several of the topics: networking, resources, life style, social life, and child care. Our pilot study showed that a cut point of 7 or more problems out of 33 possible would select the 25–30 % with most problems, and problems within several of the mentioned areas.

Training of all participating GPs may have affected their normal consultations. Their treatment might have a possible spin-over effect on the GPs’ patients with no preventive consultation; the mean weight loss of 1.6 kgs may point in that direction, but it might also be an effect of completing the baseline questionnaire.

### Strengths

This investigation was designed to be patient-centred, with a focus on the patient’s own goal for improved quality of life during the coming year [20]. The baseline questionnaire primed the patients to think in very broad terms, encompassing relationships, work problems, psychological problems, economy, social problems, problems with children, lifestyle, and health and illness behaviour. The intervention group discussed resources and barriers for achieving their prioritised goal. Our intervention fulfils the criteria of life coaching as it was based on the patient’s agenda and reflected the patient’s present wishes and needs. The dialogue was holistic, individualised, without fixed agenda. It was conducted face-to-face by GPs with special training [20, 22]. In that way, we supported the subjects’ feelings of competence, autonomy and relatedness, increasing their motivation and facilitating their self-determination. The goal and self-efficacy was supported by writing down both resources, barriers and time schedule for change [20, 26].

We collected baseline information on all included patients and dropouts, enabling us to compare the completers and the dropouts in detail without finding any statistical differences [27]. Another strength of this investigation is that the training program for the participating GPs has been well described [20, 21].

We selected two outcomes, 1-year weight loss (yes or no) (Tables 2 and 3) and extent of the weight loss (Tables 2 and 4) for our analysis of predictive factors for weight loss. Both outcomes are relevant in daily clinical work. The two most important predictors identified here, “considered short term weight loss before the preventive consultation” and “having weight loss as a prioritised goal for the next year during the preventive consultation,” seem to be robust because they were significantly related to weight loss in non-adjusted analysis, in logistic regression, and in linear regression models. These findings should be confirmed in other studies.

### Comparisons with other studies

Many GPs find the over-weight and obesity problem challenging for several reasons. In 2004, Bramlage et al. [28] reported that GPs’ recognition of over-weight (20–30 %) and obesity (50–65 %) was low, that primary-care management of over-weight and obesity was largely deficient, that doctors put forth inefficient efforts to intervene, and that patients had poor acceptance of such interventions and dissatisfaction with existing lifestyle-modification strategies. They found men had higher BMI, and obesity was more prevalent in older age groups [28], as in our study.

In several randomised studies on lifestyle counselling, weight loss after 1 year was small [15, 29, 30]. In 2011, Wadden et al. [29] reported a difference of 1.1 kgs after 1 year and 1.2 kgs after 2 years. A meta-analysis of 46 trials found a change of approximately −0.1 BMI units per month from 3 to 12 months of active programs and a regain of 0.02 ~ 0.03 BMI units per month during subsequent maintenance phases [30].

We identified in our study weight loss both in the group with preventive consultation and in the group without [20], which may be due to the combined effects of completing the baseline questionnaire, training of the GPs, and regression toward the mean weight. The additional weight loss of 3.1 kgs to a total of 4.7 kgs in the preventive consultation group with weight loss as a prioritised goal (Table 2) is interesting because it illustrates the importance of the focused discussion with GPs on resources and barriers for reaching the patients’ weight loss goals. Important elements of motivational interviewing were used to focus on supporting specific self-efficacy, which means the confidence in own ability to reach a specific goal [16, 17]. There were no specific advice given regarding food and exercise, but general advices focused on possible benefits of weight loss. An observational cohort study on the effect of a commercial weight loss program from Sweden found in an adjusted analysis that a low-calorie diet group lost 2.8 kgs more than the restricted normal-food group, and that the very low-calorie diet group lost 3.8 kgs more; dropout rates were 23 %, 26 %, and 18 %, respectively, in the three groups after 1 year [31].

Recently, Shikany et al. [32] compared a portion-controlled, nutritionally balanced, low-fat weight loss plan with a reduced energy, food-based diet. After 1 year, the weight reductions were 4.7 and 1.9 kgs, respectively, in the two groups [32]. A trend to regain the lost weight after the intervention was also observed. A much more intensive intervention with 42 sessions in obese type 2 diabetes patients was compared with three education sessions; the intensive intervention resulted in 8.6 kgs weight loss compared with 0.7 kg after 2 years [33].

Several investigations have focused on the importance of GPs’ optimism and support with regard to weight loss [34] as well as GPs’ lack of support. Wadden et al. [35] reported that nearly half of the participants in a university clinic obesity trial said that their physician had not recommended any of 10 common weight loss methods. GPs believed obesity was an important problem and used mostly brief, targeted low-intensity counselling in the face of limited patient motivation and lack of resources to support weight loss. This may reflect clinicians’ self-assessment of their ineffectiveness in this area [36]. The GPs in our study had an agenda for counselling, namely listening to the patient and discussing resources and barriers to the expressed goal for the coming year (weight loss was the most commonly chosen goal), and they had scheduled a 1-hour life-coaching consultation for a patient-centred discussion with 20 mins’ follow-up within 3 months [20].

We are intrigued by our finding that pre-interventional consideration of weight loss within 30 days was an important predictor of weight loss compared with time limits of 6 months or 12 months. This observation may emphasize the importance of respect for the readiness of the patient in health-preventive consultations. A study by Elfhag and Rössner showed that rapid initial weight loss is a predictor of success in obesity treatment [37]. A recent systematic review of 45 trials on weight loss found that behavioural interventions focusing on both food intake and physical activity were effective, with an average difference of 1.56 kgs after 1 year [38]. After 1 year, we detected the same weight loss in our control group who completed a 23-pages baseline questionnaire and had no preventive consultation.

A study from Norway supported the observation that relatively rapid weight loss (12 weeks) strongly predicts weight loss after 1 year [39]. In linear multiple regression analyses, occupational status, older age, and low mental health-related quality of life were associated with weight loss [39]. Our study confirms that the psychosocially disadvantaged participants experienced more weight loss than participants with few problems and a high-school education (Tables 2 and 3). This observation underscores that our participants with many psychosocial problems took advantage of the individual life-coaching method with elements of motivational interviewing [20].

Our study design and organisation facilitated the motivational interviewing process. This person-centred structure is supposed to have been critical to revealing the patient’s readiness for change.

## Conclusions

Participants who had a preventive consultation and identified weight loss as a prioritised goal had an average weight loss of 4.7 kgs compared to 1.6 kgs in participants without a weight loss goal or those without a preventive consultation. Consideration of weight loss within 30 days at baseline and having a preventive health consultation with weight loss as a prioritised goal for the coming year were two important predictors of weight loss after 1 year. We suggest that these two factors are important indicators of the patient’s readiness or motivation for change, and therefore should be in focus when health-related behavioural change is desired; other predictors of weight loss were having many psychosocial problems, female gender and obesity.

By structured intervention with focus on the patients self-chosen goal (weight loss), and on resources and barriers for reaching the goal, significant changes can be obtained; especially in participants with many problems, who often do not accept or drop out from screening procedures with focus on risks. In this way, general practice may contribute to bridge the gap in inequality in health.

## Abbreviations

ANOVA: ANalysis Of VAriance
BMI: body mass index
Ci: confidence interval
Cm: centimetre
GP: general practitioner
Kgs: kilogram
M^2^: height in meter squared
MCS-SF12: mental component score - short form 12 questions
N: number
OR: odds ratio
PCS-SF12: physical component score—short form 12 questions
RR: relative risk
